# Expression of Proteolytic Enzymes by Small Cell Lung Cancer Circulating Tumor Cell Lines

**DOI:** 10.3390/cancers11010114

**Published:** 2019-01-19

**Authors:** Barbara Rath, Lukas Klameth, Adelina Plangger, Maximilian Hochmair, Ernst Ulsperger, Ihor Huk, Robert Zeillinger, Gerhard Hamilton

**Affiliations:** 1 Department of Vascular Surgery, Medical University of Vienna, A-1090 Vienna, Austria; barbara.a.rath@meduniwien.ac.at (B.R.); adelina.plangger@toc.lbg.ac.at (A.P.); ihor.huk@gmail.com (I.H.); 2 Center for Pathophysiology, Infectiology and Immunology, Medical University of Vienna, A-1090 Vienna, Austria; lukas.klameth@gmail.com; 3 Respiratory Oncology Unit, Otto Wagner Hospital, A-1140 Vienna, Austria; maximilian.hochmair@wienkav.at; 4 Hospital Horn, A-3580 Horn, Austria; ernst.ulsperger@aon.at; 5 Molecular Oncology Group, Department of Obstetrics and Gynecology, Comprehensive Cancer Center-Gynecological Cancer Unit, Medical University of Vienna, A-1090 Vienna, Austria; robert.zeillinger@meduniwien.ac.at

**Keywords:** small cell lung cancer, circulating tumor cells, proteases, MMP-9, cathepsin S, metastasis

## Abstract

Small cell lung cancer (SCLC) is an aggressive type of lung cancer which disseminates vigorously and has a dismal prognosis. Metastasis of SCLC is linked to an extremely high number of circulating tumor cells (CTCs), which form chemoresistant spheroids, termed tumorospheres. Intravasation and extravasation during tumor spread requires the activity of a number of proteases to disintegrate the stroma and vascular tissue. Generation of several permanent SCLC CTC lines allowed us to screen for the expression of 35 proteases using Western blot arrays. Cell culture supernatants of two CTC lines, namely BHGc7 and 10, were analyzed for secreted proteases, including matrix metalloproteinases (MMPs), ADAM/TS, cathepsins, kallikreins, and others, and compared to proteases expressed by SCLC cell lines (GLC14, GLC16, NCI-H526 and SCLC26A). In contrast to NCI-H526 and SCLC26A, MMP-9 was highly expressed in the two CTC lines and in GLC16 derived of a relapse. Furthermore, cathepsins (S, V, X/Z/P, A and D) were highly expressed in the CTC lines, whereas ADAM/TS and kallikreins were not detectable. In conclusion, SCLC CTCs express MMP-9 and a range of cathepsins for proteolysis and, aside from tissue degradation, these enzymes are involved in cell signaling, survival, and the chemoresistance of tumor cells.

## 1. Introduction

Metastasis describes the dissemination of cancer cells from the primary tumor to adjacent normal tissue, and further to distal organs where the secondary lesions are a major cause of mortality [1,2]. Metastasis progresses in a series of discrete and interrelated steps: cancer cells are released from the primary tumor, intravasate into the blood and lymphatic systems, survive in the circulatory system, and finally extravasate at distal microvasculature and invade distant organs [3]. Metastatic cells also manipulate the microenvironment to promote the proliferation, angiogenesis, and protumor activities of normal stromal cells. The metastatic process is inherently of low efficacy, but eventually renders the cancer incurable. Cancer dissemination can start early in tumorigenesis, preceding the clinical manifestation of tumors for years [4]. In solid tumors, this implies cellular migration, movement, degradation of the extracellular matrix (ECM), and the dissolution of cell–cell contacts to neighboring epithelial cells [5,6]. Furthermore, ECM remodeling contributes to cancer progression through activation of signaling pathways, which results in invasion of single cells or clusters [7].

Circulating tumor cells (CTCs) are the primary effectors of metastatic relapse in patients with cancer, as shown by their correlation to prognosis and drug response [3,8]. Although large numbers of CTCs enter the circulatory system, only a small fraction of these cells survive successfully and extravasate into distant sites [9]. Extravasation seems to require factors altering vascular permeability and vascular endothelial barriers, including vascular endothelial growth factor VEGF, disintegrin, and metalloproteinase domain-containing proteins (ADAMs), matrix metalloproteinases (MMPs), as well as other enzymes and growth factors [10]. The cellular biological characteristics of CTCs are difficult to study due to their heterogeneity, occurrence in low numbers, and due to the inability to select for the actual metastasis-inducing subpopulation which will ultimately survive and generate secondary tumors [3,4]. Therefore, random isolation of single cells and tests for markers, secreted enzymes and cytokines are not suitable for characterizing the properties of truly effective CTCs. However, the blood of small cell lung cancer (SCLC) patients can exhibit extreme numbers of CTCs and the circulation of a sufficient number of CTCs with metastasis-initiating potential allowed us to establish several permanent CTC lines ex vivo [11,12,13].

Lung cancer remains one of the most prevalent and malignant cancers worldwide, with SCLC representing its most aggressive variant [14,15]. The majority of cases are diagnosed at late stages, when local invasion and placement of distal metastases has already occurred. SCLC dissemination is known to occur via three major routes, namely blood, lymphatic vessels, and transcoelomic spread into the pleural, pericardial, and abdominal cavities [14,15]. Involved steps comprise angiogenesis, degradation of ECM by proteases, increases in cellular motility and resistance, as well as protection from immune surveillance [16,17]. The process of cancer metastasis and the mechanisms dictating cancer dissemination and setup of secondary lesions is still poorly understood [17].

SCLC represents a suitable model for studying early tumor spread and the development of drug resistance. This tumor entity is distinguished by an extremely high count of CTCs, which was reported to be linked to prognosis and response to therapy [13]. SCLC CTCs enriched from blood samples of patients with more than 400 CTCs/7.5 mL blood could be used to establish xenotransplants in immunocompromised mice [18,19]. However, an investigation of the proteases of CTCs employed to dissolute ECM would depend on the availability of a larger number of a homogenous and pure population of relevant tumor-initiating cells. So far, cultures of CTCs, except our SCLC CTC lines, were only reported for one colon cancer and several breast cancer CTC lines [20,21]. Cultures of patient-derived CTCs may allow the study of mechanisms of tumorigenesis, invasion, and metastasis and novel therapeutic strategies [22]. Our CTC lines were established from patients with metastatic disease and showed similar characteristics and formation of highly chemoresistant spheroids, termed tumorospheres [23]. In the present work, we used two CTC cell lines, namely BHGc7 and BHGc10, which are tumorigenic in NOD-SCID mice, to screen for the expression of 35 proteases using Western blot arrays. Proteases are involved in tumor angiogenesis, invasion and metastasis during malignant progression and this group comprises the largest family of enzymes in the human genome [5,6]. They are a part of a system of proteolytic interactions between factors of the tumor microenvironment and proteases—such as cathepsins, urokinase-type plasminogen activator (uPA), and several matrix metalloproteinases (MMPs), among others. Besides the first attempt to identify proteases expressed by SCLC CTCs lines, we included a cell line pair, GLC14 and GLC16, established before and after chemotherapy, as well as two cell lines derived from a bone metastasis, NCI-H526, and from a pleural effusion, SCLC26A, as representatives of local metastatic lesions [24,25]. Additionally, proteases were screened in a conditioned medium of a coculture of SCLC CTC and macrophages, which are recruited and educated by such tumor cells [26]. Functionally, the invasion of the SCLC CTC cells into ECM was tested.

## 2. Results

### 2.1. Protease Expression of GLC14 and GLC16 Cell Lines

**Figure 1:**
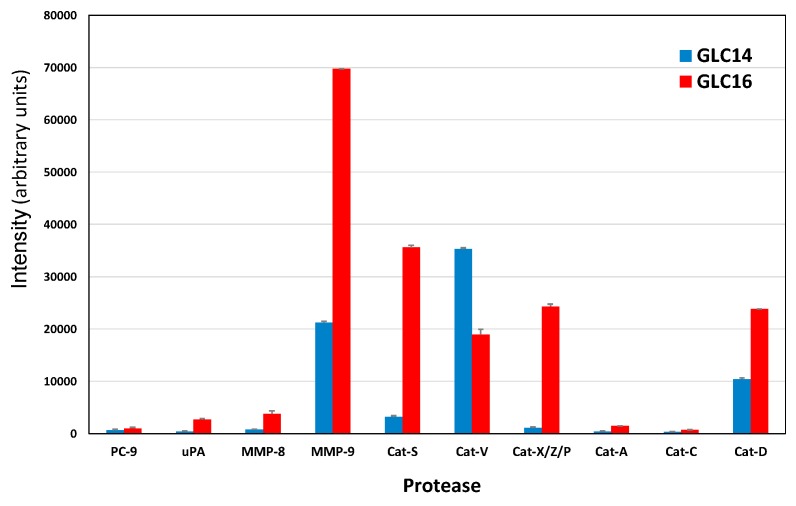
Selected proteases expressed by GLC14 and GLC16 small cell lung cancer (SCLC) cell lines, established from the same patient before therapy and following relapse, respectively. Values represent mean ± SD (arbitrary intensity units) and all differences are statistically significant, except for PC-9 and Cat-C.

For all of the following experiments, only the significantly expressed proteases out of the 35 enzymes assayed are shown. GLC14 and GLC16 represent lines established from metastases of the same patient before and after failure of chemoradiotherapy, respectively. Results demonstrate that during the progressive disease, GLC16 showed elevated expression of most proteases, with marked overexpression of MMP-9, cathepsins S, X/Z/P and D, as well as partial downregulation of cathepsin V (Figure 1).

### 2.2. Protease Expression of NCI-H526 and SCLC26A Cell Lines

**Figure 2:**
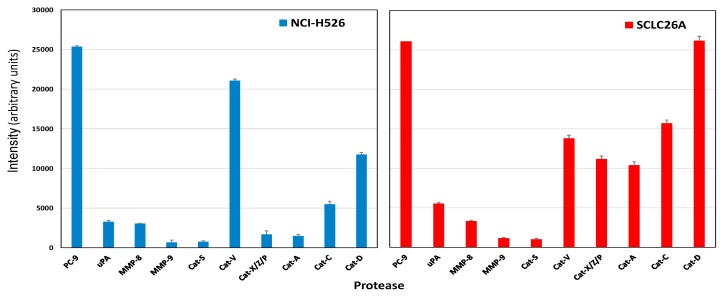
The figure shows selected proteases expressed by NCI-H526 (left side) and SCLC26A (right side) SCLC cell lines, established from a bone metastasis and from pleural effusion, respectively. Values represent mean ± SD.

NCI-H526 and SCLC26A represent local metastases to bone and pleural fluid, respectively. NCI-H526 exhibited high expression of proprotein convertase-9 (PC-9) and cathepsins V and D, with weaker occurrence of cathepsin C and low levels of other cathepsins, urokinase-type plasminogen activator (uPA), and MMP-8/9 (Figure 2, left side). The SCLC26A cell line again highly expressed PC-9 and cathepsins V, X/Z/P, A, C, and D (Figure 2, right side). Secreted MMP-8, uPA, and cathepsin S were present at very low concentrations.

### 2.3. Protease Expression of CTC Cell Lines BHGc7 and BHGc10

**Figure 3:**
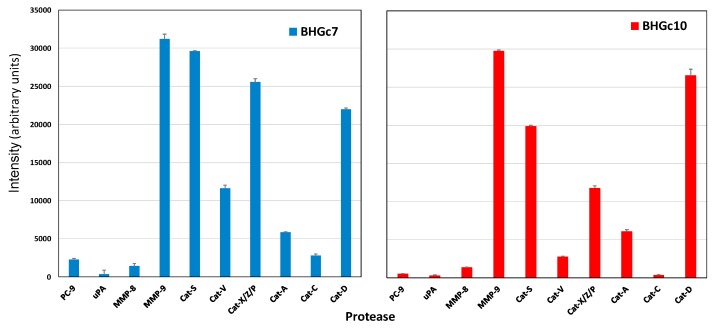
Selected proteases expressed by BHGc7 (left side) and BHGc10 (right side) SCLC CTC lines, established from blood samples of two refractory SCLC patients, respectively. Values represent mean ± SD.

The two CTC cell lines derived from SCLC shared expression of MMP-9 and cathepsin S, the latter not found in the other cell lines tested, except in metastatic GLC16. Furthermore, cathepsins V, X/Z/P, A, and D were maintained in BHGc10, and all cathepsins tested, namely V, X/Z/P, A, C, and D in BHGc7 (Figure 3). PC-9 was not expressed in significant concentrations in both CTC cell lines, as well as uPA, MMP-8 and all other of the 35 proteases included in the test panel.

### 2.4. Protease Expression of a Macrophage CTC Coculture Supernatant

**Figure 4:**
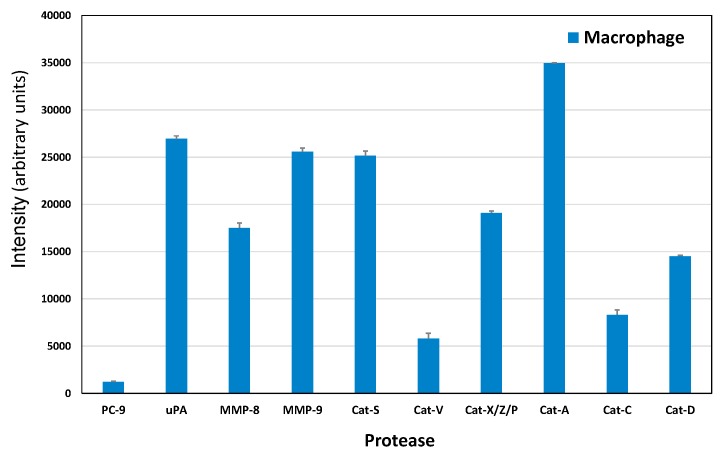
Proteases expressed by macrophages corresponding to the enzymes detected in SCLC lines. Values represent mean ± SD.

Macrophages which developed in coculture with the BHGc10 CTC line were supplemented with fresh medium, and following incubation tested for the presence of proteases using the Western blot arrays. The result showed that this type of macrophage expressed all proteases found in SCLC CTCs in abundance, with the addition of high amounts of uPA and MMP-8 (Figure 4).

### 2.5. ECM Invasion of SCLC CTC Cells

**Figure 5:**
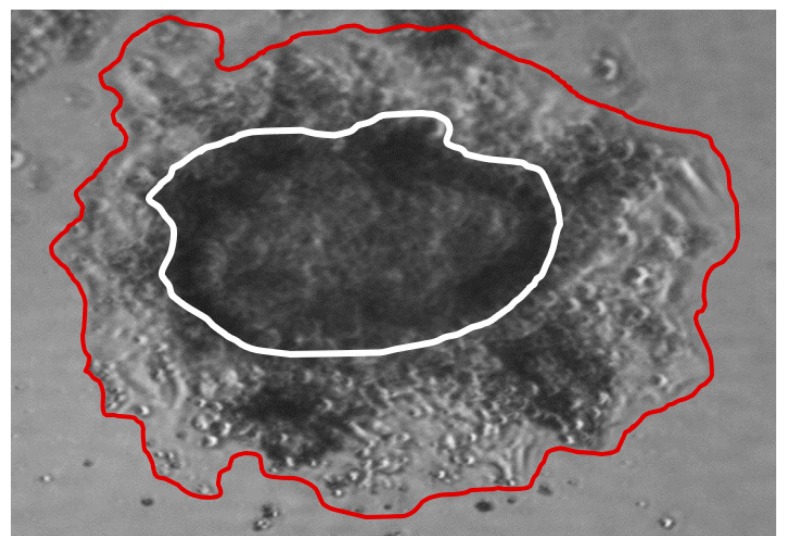
Light microscopic picture of a SCLC tumorosphere embedded in extracellular matrix (ECM) showing invasive outgrowth of cancer cells. The white line indicates the contour of the original spheroid at beginning (magnification 40×).

SCLC CTC lines grow as large spheroids, called tumorospheres, which continuously increase in diameter in regular tissue cultures. Upon embedding in Matrigel, cells of the tumorosphere started to invade the surrounding matrix (Figure 5). According to the light microscopic appearance, the ECM was dissolved during this invasive growth. Quantitation of the areas covered by the original cluster (60,070 square pixels) and the invaded cells (142,460 square pixels) revealed an approximately 2.4-fold extension of the initial tumorosphere within 12 days. The pan-cathepsin inhibitor E-64 did not inhibit invasion into ECM (data not shown), pointing to MMP-9 as major effector.

### 2.6. Scheme of Tumor Cell and CTC—Normal Cells Protease Interaction

**Figure 6:**
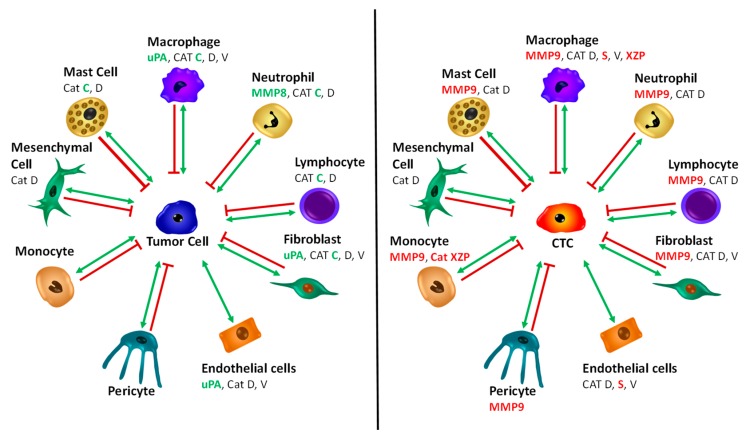
Scheme of involvement of distinct proteases in tumor cell-normal cell and circulating tumor cells (CTC)-normal cell interactions.

Figure 6 depicts the MMP and cathepsin proteases involved in tumor cell and CTC interactions with normal cell types. Proteases occurring in tumor cell interactions are shown in green color, and those in CTC interactions in red color. For CTCs, the specific proteases comprise MMP9, Cathepsin S, and cathepsin X/Z/P. Cathepsin C is involved in tumor interactions with mast cells, lymphocytes, fibroblasts and macrophages, and uPA with fibroblasts and endothelial cells, respectively. Additionally, MMP-8 functions in relation to neutrophils. In the case of CTCs, the cells interact with all normal cell types, except mesenchymal cells via one or more of the proteases specifically expressed.

## 3. Discussion

Tumor cell spread to distant sites is a complex process involving multiple cell types, soluble growth factors, adhesion receptors, and tissue remodeling [1,27]. Pericellular proteases are involved in cancer invasion and metastasis due to their ability to degrade ECM constituents [11,12]. Furthermore, proteases regulate progression and dissemination through processing of cell adhesion molecules, cytokines, growth factors, and kinases [1]. SCLC is surrounded by an extensive stroma of ECM, protecting cancer cells by prosurvival signaling [28,29,30]. Although several enzymes of the proteolytic tumor network are associated with invasion and metastasis, the proteases responsible for the migration and invasion of CTCs have not been identified so far. CTCs are highly heterogeneous and only a small fraction of these cells is capable of inducing metastases [9,15]. Availability of two CTC cell lines established from SCLC enabled us to screen the proteases secreted by these tumor cells in vitro. Both CTC lines used were in tissue culture for several months after initiation of the lines. However, according to their transcriptomic and proteomic profile as well as biomarkers and morphology (formation of spheroids), these lines exhibit a stable phenotype. This Western blot screen comprised 35 proteases including ADAMs 8, 9, S1, and S13; cathepsins A, B, C, D, E, L, S, V, and X/Z/P; MMPs 1, 2, 3, 7, 8, 9, 10, 12, 13; kallikreins 5, 6, 7, 10, 11, 13; neprilysin/CD10, presenilin-1, PC-9, proteinase 3, and uPA. Of all these enzymes, uPA, MMP-8 and -9 as well as several cathepsins were expressed in SCLC tumor lines, the two SCLC CTCs and conditioned macrophages in our screening experiments.

Longitudinal biopsies are rarely available for SCLC patients. However, a series of three cell lines, namely GLC14, GLC16, and GLC19, were established from the biopsies of a single SCLC patient [24]. In detail, the GLC14 cell line was from a right supraclavicular node metastasis of the patient and, following treatment with several cycles of cyclophosphamide, doxorubicin, and etoposide, the chemoresistant GLC16 cell line was established from a biopsy of the relapsing tumor [31,32]. Our results demonstrate that progression to this chemoresistant relapse is characterized by increased expression of MMP-9, as well as cathepsins S, X/Z/P, D and decreased expression of cathepsin V. In order to study protease expression by locally invasive tumor cells, we employed cell lines NCI-H526 and SCLC26A, representing a bone metastasis and a local pleural metastasis, respectively. Both cell lines exhibited high expression of PC-9. The PCs are secretory proteolytic enzymes that activate precursor proteins into biologically active forms by limited proteolysis at internal sites [33]. Many PC substrates are well known cancer-associated proteins such as growth factors, growth factor receptors, integrins, and MMPs [34]. For example, insulin-like growth factor 1 (IGF-1) and its receptor, transforming growth factor beta (TGF-beta), VEGF-C, and MMPs have direct roles in tumor progression and metastasis [35]. Additionally, IGF-1 and platelet-derived growth factor (PDGF) were found to mediate a mitogenic/antiapoptotic function through Akt activation [36]. SCLC26A was found to rely on EGF, IGF-1, and insulin for proliferation, thus, PC-9 seems to be required to process growth factors in these two metastatic cell lines (results not shown). 

Protease secretion of two CTC cell lines BHGc7 and BHGc10, derived from SCLC, is largely confined to MMP-9 and several members of the cathepsin family, with cathepsin S exclusively found in the CTCs and the metastatic and chemoresistant GLC16 cell line, but not in lines established from local metastases. Cathepsins V, X/Z/P, A, and D are expressed in BHGc10, and all cathepsins tested, namely V, X/Z/P, A, C, and D in BHGc7. PC-9 is not expressed in significant concentrations in both CTC cell lines, as well as uPA, MMP-8 and all other of the 35 proteases included in the Western blot array panel. In most cancers, there are increased levels of one or several members of the MMPs and, in particular, MMP-9 is closely associated with the invasive and metastatic potential of most types of solid cancers [37,38,39]. MMP-9 is expressed by neutrophils, macrophages, fibroblasts, and endothelial cells, among others, and can cleave many ECM proteins, soluble mediators and release cell surface proteins. The most important substrates of this enzyme are gelatin, collagen, elastin, and type-IV collagen of basement membranes [37,38,39]. In lung cancer, both non-small cell lung cancer NSCLC and SCLC tumor samples showed significantly higher MMP-9 expression compared to normal tissues as well as elevated MMP-9 in serum samples [40,41]. MMPs and tissue inhibitors of metalloproteinase (TIMPs) are widely expressed in SCLC [42,43]. MMP-9 was found to be elevated in the serum of NSCLC patients compared to healthy controls and to potentiates formation of pulmonary metastasis [44]. Furthermore, the MMP-9 serum level was higher in chemoresistant prostate cancer patients upon disease progression [45]. Among normal cells, inflammatory cell-derived MMP-9 promotes extravasation in combination with tumor-derived MMP-9 and endothelial cell clusters at metastatic sites are stimulated to produce MMP-9 by circulating VEGF [39]. Furthermore, MMP-9 from inflammatory cells, particularly neutrophils and tumor-associated macrophages (TAMs), codetermines prognosis and outcome [46]. In chronic obstructive pulmonary disease (COPD), increased expression of MMP-9 by inflammatory cells e.g., neutrophils and macrophages, is correlated with a variety of processes that cause lung damage [47]. However, the development of MMP broad-range inhibitors failed to result in a clinical benefit for patients [48].

Secretion of cathepsin S seems to be a specific characteristic of the SCLC CTCs tested. A large study showed a significant correlation between elevated serum cathepsin S levels and increased mortality risk in older adults [49]. Although cysteine cathepsins have been identified as key regulators of cancer growth, their specific role in tumor development remains unclear [50]. Cysteine cathepsin proteases are frequently dysregulated during transformation and participate in cancer progression, invasion, metastasis, and drug resistance [6,51]. The human cysteine cathepsin family comprises 11 endopeptidases which are synthesized as inactive zymogens and are activated in acidic tumor regions [52,53]. Intracellular cathepsins are acid hydrolases involved in protein catabolism, autophagy, and signal transduction. Secreted cathepsins adapt the tumor microenvironment through degradation of ECM and processing of growth factors, cytokines, and chemokines. Cathepsins contribute to tissue invasion and metastasis by cleavage of cell-cell adhesion molecules. Besides cancer cells, various other cell types express cathepsins with exceptional abundance of cathepsins B, H and S in TAMs. Furthermore, cathepsin Z is essential for the activation of focal adhesion kinase (FAK) and SRC and, furthermore, cathepsins regulate tumor angiogenesis [6,54,55,56]. For example, coadministration of the pan-cathepsin inhibitor E-64 with gemcitabine doubled the median survival in a murine model of pancreatic cancer [57]. However, the clinical failure of broad-spectrum MMP inhibitors has disapproved therapeutic strategies targeting protease families in general.

Cathepsin S is involved in presentation on major histocompatibility complex (MHC) class II molecules, and in contrast to other lysosomal proteases, it retains stability outside the lysosome and cleaves ECM proteins including laminin, fibronectin, elastin, osteocalcin, and some collagens [58]. Immune cells, including macrophages and microglia, secrete cathepsin S in response to inflammatory mediators derived from tumor cells [59]. Investigations on breast, lung, brain and head and neck tumors, as well as in body fluids of ovarian, uterine, melanoma, and colorectal carcinoma bearing patients, have shown that cathepsins are highly predictive for survival [60,61,62,63]. Cysteine cathepsins upregulation has been demonstrated in many human tumors, including breast, lung, brain, gastrointestinal, head and neck cancer, and melanoma [64]. Cathepsin S plays an active role in angiogenesis by generation of proangiogenic peptides, promotes tumor growth, and has been shown to be a significant prognostic factor for patients with glioblastoma [58,65]. High cathepsin S expression at the primary site correlated with decreased brain metastasis-free survival in breast cancer patients [61]. Both macrophages and tumor cells produce cathepsin S, and only the combined depletion significantly reduced brain metastasis in vivo. Sevenich et al. described a role for cathepsin S in brain-specific metastasis and identified JAM-B, a blood–brain barrier component, as a cathepsin S substrate [61]. A cathepsin S inhibitor reduced MC38 and MCF7 tumor cell invasion and furthermore, significantly reduced vascular endothelial tubule formation in vitro [62]. The inhibitor reduced the tumor growth of both cell lines in an in vivo xenograft model. The initial development of cathepsin S inhibitors targeted irreversible, covalent inhibitors, but more recently, the focus has been on reversible inhibitors [66]. The specific expression of cathepsin S by SCLC CTCs seems to be of high significance, since SCLC frequently leads to development of brain metastases, which continue to be associated with short median survival of 4.9 months [67]. Cathepsin D is a protease involved in the metastasis and angiogenesis of mammary carcinomas [68]. Procathepsin D (pCD) is overexpressed and secreted by cells of various tumor types, including breast and lung carcinomas, affecting multiple features of tumor cells including proliferation, invasion, metastasis, and apoptosis [69]. Studies have demonstrated that enzymatic function of cathepsin D is not restricted solely to acidic milieu of lysosomes, with important consequences in regulation of apoptosis [70]. Apoptosis is also regulated by catalytically inactive mutants of cathepsin D, which suggests that it interacts with other important molecules and influences cell signaling. Moreover, procathepsin D (pCatD), secreted from cancer cells, acts as a mitogen on both cancer and stromal cells, and stimulates their pro-invasive and pro-metastatic properties.

Despite the role of uPA and its receptor uPAR/CD87 as major regulators of ECM degradation, and their involvement in cell migration and invasion under physiological and pathological conditions, they were not found in the CTCs tested here [71]. Otherwise, this system is involved in the development of most invasive cancer phenotypes and is a strong predictor of poor patient survival [72]. Furthermore, high serum uPAR(I) levels are associated with short overall survival in SCLC patients and identify chemoresistant cells [71,73]. Endopeptidase CD10 hydrolyzes bioactive peptides, including neuropeptides, but was not found in our assays, in accordance with previous findings demonstrating the absence of CD10 in most SCLCs [74,75]. DPPIV/CD26 is expressed in almost all cases of adenocarcinoma, whereas all cases of squamous cell carcinoma, SCLC, large cell carcinoma and carcinoid were negative [76,77]. ADAM/TSs are involved in the regulation of growth factor activities and integrin functions, leading to promotion of cell growth and invasion [78,79,80]. ADAM8 is overexpressed in the vast majority of lung cancers and can be a diagnostic marker of lung cancer [81,82]. Extensive cytoplasmic expression of tissue and plasma kallikrein was observed in SCLC and NSCLC, but these enzymes as well as ADAM/TS were not detected in BHGc7/10 [83].

The functional activity of CTC-derived proteases has been studied with help of a microfluidics system [84]. This system concentrates rare cancer cells by size, flushes the CTCs to remove contaminants, and encapsulates the CTCs into microdroplets containing a fluorescent MMP substrate. CTCs from prostate cancer patients showed increased MMP activity (1.7- to 200-fold) over those of leukocytes from the same patient (average ratio 2.6 ± 1.5). Samples from 6/7 metastatic castration-resistant prostate cancer patients contained CTCs, and 87% of these CTCs secreted MMPs. However, this contradicts the finding that only a very small fraction of CTCs are actual metastasis-initiating cells. Nevertheless, our results are in agreement with this microfluidic study which proved increased MMP-9 activity. Embedding of the SCLC CTC clusters and subsequent cellular outgrowth demonstrates invasion, and is expected to provide a suitable model for studying the participation of individual proteases. In a suspension tissue culture, the same clusters increase in size continuously but show no release of viable cells [23]. In conclusion, assessment of the pattern of secreted proteases of SCLC CTCs revealed for the first time cathepsin S as specific enzyme associated with this class of unique cells, executing tumor dissemination to distal sites. Cathepsin S has been discussed as putative cancer target, but not in relation to CTCs [85]. Specific cathepsin S inhibitors like LY3000328 have been developed for application in nonmalignant diseases, and may be checked for their effects on tumor spread [86,87].

## 4. Materials and Methods

### 4.1. Cell Lines and Tissue Culture

GLC14 and GLC16 were obtained from Department of Radiation Biology, the Finsen Centre, National University Hospital, Copenhagen, Denmark and NCI-H526 was obtained from the American Tissue Culture Collection (ATCC, Rockville, MD, USA). GLC14, GLC16, and GLC19 constitute a series of three cell lines which have been established from biopsies of a single SCLC patient [24]. In detail, the GLC14 cell line was from a right supraclavicular node metastasis of the patient and, following treatment with several cycles of cyclophosphamide, doxorubicin, and etoposide, the chemoresistant GLC16 cell line was established from a biopsy of the relapsing tumor [31,32]. SCLC26A was established in our laboratory from pleural effusion of an SCLC patient before treatment and the two CTC cell lines, BHGc7 and BHGc10, were grown from peripheral blood samples of two refractory SCLC patients [10]. Cell lines were cultured in RPMI-1640 (Sigma-Aldrich, St. Louis, MO, USA) medium supplemented with 10% fetal bovine serum (Seromed, Berlin, Germany) and antibiotics (Sigma-Aldrich, penicillin-streptomycin-neomycin solution). All cell lines were grown in suspension or loosely attached and were subcultivated by replacing part of the medium. All other reagents were from Sigma-Aldrich.

### 4.2. Western Blot Protease Screening Array

For assessment of the proteases expressed, cell culture supernatants were processed using a Human Proteome Profiler Protease Kit according to the manufacturer’s instructions (R&D Systems, Minneapolis, MN, USA). In brief, this Western blot array comprised reagents to detect 35 proteases, including ADAM/TS, kallikreins, MMPs, cathepsins, uPA, neprilysin (CD10), presenilin-1, DPPIV (CD26), and proprotein convertase 9 (PC-9). Assays were performed in duplicate. The different arrays contain several control spots to calibrate for protein content of the samples applied. Conditioned medium of the respective cell lines (500 µL) were used for performing the assay and the spots detected by chemoluminescence were analyzed using Origin 9.0 software (OriginLab, Northampton, MA, USA).

### 4.3. ECM Invasion Assay

Tumorospheres of BHGc10 cell line were isolated by sedimentation and resuspended in Matrigel (Sigma-Aldrich). Matrigel was thawed overnight at 2–8 °C before use, mixed with medium containing the spheroids (1:1) and dispensed to 18-well plates (Greiner, Kremsmuenster, Austria) using pre-cooled pipettes. Outgrowth of tumor cells was observed by light microscopy and areas covered by the original cluster and the invaded cells quantitated using Image J.

### 4.4. Statistics

Results were evaluated using unpaired *t* tests, using Origin 9.0 software. *p* < 0.05 was regarded as statistically significant.

## 5. Conclusions

Due to the high heterogeneity of the CTCs, detection of the expression of proteases has been limited to demonstration of increased levels of MMP-9 compared to normal blood cell types [84]. Our screens have shown the expression of MMP-9 and cathepsins by pure populations of CTC lines for the first time. This analysis has allowed for the differentiation of the protease expression of tumor and normal cells, respectively [88,89]. Detection of Cathepsin S in SCLC CTCs may be of special importance for this tumor in respect to frequent occurrence of brain metastases. Invasion of ECM by SCLC CTCs may constitute an important model for studying the participating cellular factors.
